# Characteristics of gastrointestinal symptoms and function following endoscopic submucosal dissection and treatment of the gastrointestinal symptoms using rikkunshito

**DOI:** 10.3892/etm.2013.1299

**Published:** 2013-09-13

**Authors:** RYOHEI UEHARA, HAJIME ISOMOTO, HITOMI MINAMI, NAOYUKI YAMAGUCHI, KEN OHNITA, TATSUKI ICHIKAWA, FUMINAO TAKESHIMA, SABURO SHIKUWA, KAZUHIKO NAKAO

**Affiliations:** Department of Gastroenterology and Hepatology, Nagasaki University Graduate School of Biomedical Sciences, Nagasaki 852-8501, Japan

**Keywords:** endoscopic submucosal dissection, gastric cancer, gastric emptying, rikkunshito, abdominal pain

## Abstract

The aim of the present study was to investigate the gastrointestinal (GI) symptoms and gastric emptying following endoscopic submucosal dissection (ESD), as well as to evaluate a novel treatment strategy using rikkunshito, a traditional Japanese herbal medicine. GI symptoms and gastric emptying were evaluated 6–8 days after ESD as part of the Step I study. In the Step 1 study, the Gastrointestinal Symptom Rating Scale (GSRS) scores of the two groups after 4 and 8 weeks of treatment with either a proton pump inhibitor (PPI; PPI monotreatment group, n=5) or a PPI plus rikkunshito (PPI + rikkunshito group, n=8) were compared against baseline values. Abdominal pain and constipation occurred in the majority of patients after ESD. The mean T-max 6–8 days after gastric emptying was 75.4±13.6 min, which was significantly longer compared with that reported in healthy subjects (43.9±10.3 min). In the Step 2 study, the total GSRS score was significantly improved only in the PPI + rikkunshito group after 8 weeks of treatment. In conclusion, ESD affects gastric emptying and is associated with an increased incidence of upper GI symptoms such as abdominal pain and indigestion. Rikkunshito may be useful as a novel supporting therapeutic drug for the treatment of GI symptoms in patients who have undergone ESD.

## Introduction

Endoscopic mucosal resection (EMR) is widely used as an endoscopic therapy for gastric cancer (1). However, EMR is limited in resection size and, therefore, piecemeal resection is performed in cases of large lesions resulting in an imprecise histological evaluation and in a high frequency of local recurrence (2). Endoscopic submucosal dissection (ESD) is a novel endoscopic technique that enables the en bloc resection of large superficial gastric cancers (3). In a multicenter retrospective study of endoscopic resection for early gastric cancer (EGC) (4), the 3-year cumulative residual/recurrence-free rate following ESD was higher compared with that of EMR. Regarding the overall survival rate of patients subjected to ESD, we previously demonstrated that the 5-year overall survival rate was 97.1% in patients with lesions that fulfilled the standard guideline criteria and 97.2% in patients with EGC that did not meet the guideline criteria but fulfilled the expanded inclusion criteria (5). In addition, ESD not only reduces the surgical risk and the recurrence rate of gastric cancer, but also improves the quality of life (QOL) of patients (6). Even though ESD is useful in the treatment of EGC, it has been reported that in addition to the risk of bleeding and perforation, patients subjected to ESD may also experience gastrointestinal (GI) symptoms such as belching and bloating (7). Proton pump inhibitors (PPIs) are widely used to treat ESD-induced gastric bleeding and ulcers (8,9). However, PPIs are not able to totally relieve ulcer symptoms and upper abdominal discomfort when the ulcer is large (8,9). Since the GI symptoms and gastric motor functions following ESD have been fully elucidated, it has become possible to manage the clinical condition of a patient after ESD. In the present study, we initially investigated the characteristics of GI symptoms and gastric emptying following ESD.

Rikkunshito, a traditional Japanese medicine, is widely used to treat upper GI symptoms such as gastroesophageal reflux (10), dyspeptic symptoms (11) and chemotherapy-induced nausea (12). Rikkunshito has been reported to have a dual action on the stomach, including relaxation of the proximal stomach (13) and contraction of the distal stomach (14). Thus, since rikkunshito may improve the QOL of patients subjected to ESD, we also investigated the effects of a PPI alone (PPI monotreatment group) and a PPI combined with rikkunshito (PPI + rikkunshito group) on GI symptoms following ESD in the present study, .

## Patients and methods

### Subjects

A total of 33 patients with gastric cancer (mean age 70 years; male/female, 25/8) who had undergone ESD at Nagasaki University Hospital (Nagasaki, Japan) between January 2010 and September 2011 were included in the present study as primary subjects (Step 1). The primary subjects met the following inclusion criteria: i) patients with gastric cancer subjected to ESD, ii) patients aged >20 and <81 years, iii) patients for whom oral administration was possible, and iv) patients who provided written informed consent regarding their participation in the study. The exclusion criteria were the following: i) presence of an additional type of cancer, ii) patients who needed chemotherapy, iii) patients with serious complications (liver, kidney, heart, blood or metabolic disorders), iv) patients with alcohol or drug dependence, v) patients under treatment for a psychological disease, vi) patients with ulcerative colitis, Crohn’s disease or irritable bowel syndrome (IBS), vii) women who were pregnant or wished to become pregnant during the study or the follow-up period, as well as lactating women, viii) patients who had received traditional Japanese medicine including the test drug within one month prior to the administration of the test drug, ix) patients with a history of hypersensitivity to traditional Japanese medicine including the test drug, and x) patients who were not considered eligible for inclusion in the present study by the chief investigator.

### Study design

This study (UMIN000002302) was a prospective, randomized, parallel, comparative study to examine the pharmacological effects, efficacy and safety of drug therapy in gastric cancer patients subjected to ESD. This study was conducted based on ethical guidelines for clinical studies, taking into consideration the human rights and privacy of the patients. The protocol of this study was approved by the Institutional Review Board of Nagasaki University Hospital.

### Study procedures and questionnaire

**Figure 1.**
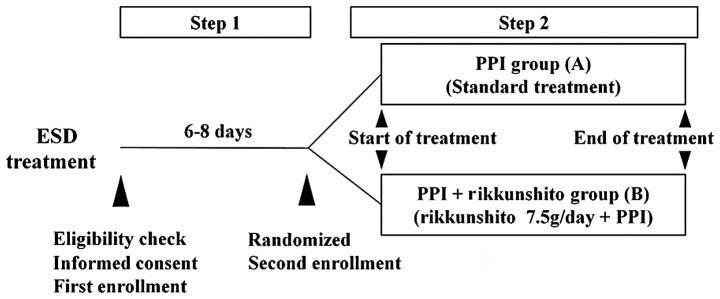
Study design. Patients who were scored ≥3 more than the average GSRS score for abdominal pain or indigestion 6–8 days after ESD were randomized to either of the two groups in the Step 2 study. ESD, endoscopic submucosal dissection; PPI, proton pump inhibitor.

The study procedures followed are shown in Fig. 1. After obtaining written informed consent regarding participation prior to ESD, patients who fulfilled all the inclusion criteria were included in this study. The GI symptoms of the patients were assessed using the Gastrointestinal Symptom Rating Scale (GSRS) 6–8 days following ESD. Gastric emptying was evaluated using the [^13^C]-labeled acetate breath test (described in detail later) that consisted of the administration of a liquid meal [OKUNOS-A (200 ml) containing ^13^C-sodium acetate (100 mg)] and the determination of the peak time of the 13C% dose-excess curve (T-max) after the evaluation of GI symptoms. The Japanese Ministry of Health, Labour and Welfare has approved rikkunshito for the treatment of abdominal pain and indigestion. Thirteen patients who scored ≥3 more than the average GSRS scores for abdominal pain or indigestion 6–8 days after ESD were included in the Step 2 study, and were randomized to the PPI group [standard treatment with rabeprazole monotreatment (20 mg/day), twice/day (b.i.d); n=5] or the PPI + rikkunshito group [rabeprazole (20 mg/day, b.i.d) combined with rikkunshito (7.5 g/day), three times/day (t.i.d); n=8]. The respective GSRS scores of the two groups after 4 and 8 weeks of treatment were compared with baseline values.

### Measurement of GI symptoms

The GI symptoms were evaluated using the GSRS, which is an inquiry table consisting of 15 items for the evaluation of general GI symptoms (15). Each GSRS item is rated on a 7-point Likert scale ranging from no discomfort to very severe discomfort. Based on a factor analysis, the 15 GSRS items break down into the following five scales: abdominal pain (abdominal pain, hunger pain and nausea), reflux syndrome (heartburn and acid regurgitation), diarrhea syndrome (diarrhea, loose stools and urgent need for defecation), indigestion syndrome (borborygmus, abdominal distension, eructation and increased flatus) and constipation syndrome (constipation, hard stools and a feeling of incomplete evacuation).

In the Step 2 study, the overall GSRS score and subscales after 4 and 8 weeks of treatment were compared against baseline values to evaluate the potential improvement of GI symptoms in each group.

### Measurement of gastric emptying

The gastric emptying test was performed according to the protocol of the ^13^C-acetate breath test standardized by the Japan Society of Smooth Muscle Research (16). Briefly, a commercially available diet (OKUNOS-A; Horika Foods, Tokyo, Japan) was used as the test meal. A 200 ml portion of the diet contained 9.8 g protein, 5.2 g fat, 28.6 g carbohydrate and 200 kcal. The test meal was enriched with 100 mg ^13^C-sodium acetate (Wako Pure Chemicals Industries, Osaka, Japan). Breath samples were collected into 300 cc packs (Otsuka Pharmaceutical Co., Tokyo, Japan). The analysis of the ^13^CO_2_/^12^CO_2_ enrichment in the breath samples was performed using an infrared spectrophotometer (UBiT-IR200; Otsuka Electronics Co., Tokyo, Japan). The T-max of gastric emptying was calculated using Excel software (Star Medical, Tokyo, Japan). The cumulative excretion of ^13^CO_2_ (as a percentage of the ingested dose) was also calculated.

Once baseline measurements had been taken after an overnight fast, subjects consumed the test meal within 15 min. Upon completion of the meal (t=0 min), sequential postprandial measurements of gastric emptying were taken. Expired breath samples were collected at t=0 min and every 5 min for the first half hour after the meal was consumed, and at 15-min intervals from t=30 min until 90 min for the detection of ^13^CO_2_.

### Adverse events, safety and tolerability

Safety and tolerability were assessed by recording all adverse events, and changes in hematological and clinical laboratory variables were measured during the screening visit and after the post-dose esophagogastroduodenoscopy. An adverse event was defined as any unfavorable or unintended sign, whether or not it was considered to be causally related to the drugs used in this study.

### Compliance

Treatment compliance was defined as the percentage of the test drug used. A treatment compliance of ≥66.6% was considered acceptable.

### Statistical analysis

The treatment response in each group was evaluated based on changes in the GSRS or GSRS sub-item scores prior to and following treatment using the Wilcoxon signed-rank test. Furthermore, the improvement rate in each subject was calculated from these scores, and the mean values were compared between the two groups using the Wilcoxon rank-sum test. This test was also used to compare background factors such as age and body mass index (BMI). The distribution of gender was compared using Fisher’s exact test. P<0.05 was considered to indicate a statistically significant difference. All data are expressed as the mean ± standard deviation (SD).

## Results

### Patient characteristics

Patient characteristics including mean age, male/female ratio, BMI and clinicopathological characteristics of EGC are shown in Table I. The standard guideline criteria and expanded criteria for ESD were established by the Japanese Gastric Cancer Association (17,18). The standard guideline criteria were defined as differentiated mucosal cancer of ≤2 cm without ulcer. The expanded criteria encompassed non-ulcerative differentiated cancer >2 cm, ulcerative differentiated cancer <3 cm, non-ulcerative undifferentiated cancer <2 cm and differentiated, submucosal invading (limited to 500 μm below the lamina propria) cancer <3 cm in diameter. In 11 patients the lesion fulfilled the standard guideline criteria, in seven patients the lesion fulfilled the expanded criteria, and in 15 patients the lesion was an adenoma or atypical duct hyper-plasia that did not comply with either the standard guideline or expanded criteria. The lesions were removed by en bloc resection in all patients. The incidence of local tumor recurrence was 3% (complete removal:incomplete removal, 32:1).

### Gastric emptying in EGC patients who had undergone ESD

**Figure 2.**
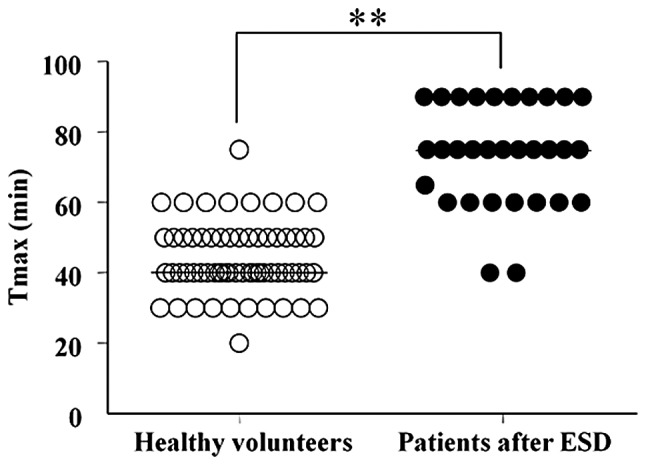
Gastric emptying in patients with early gastric cancer subjected to ESD. The reference range of T-max was derived from measurements in 63 healthy volunteers by the Japan Society of Smooth Muscle Research (16). Horizontal bars represent the median value in each group. ^**^P<0.01, healthy volunteers vs. patients with ESD (Wilcoxon rank-sum test). ESD, endoscopic submucosal dissection.

Of the 33 patients who had undergone ESD, the evaluation of gastric emptying was possible in 32 patients. The reference range of T-max was derived from 63 healthy volunteers by the Japan Society of Smooth Muscle Research; the mean ± SD of the T-max was 43.9±10.3 min (16). Gastric emptying following ESD in the patients with EGC in the present study is shown in Fig. 2. The mean T-max 6–8 days after ESD was 75.4±13.6 min which was significantly increased compared with that in healthy subjects (43.9±10.3 min).

### Incidence of GI symptoms according to location of the lesion

**Figure 3.**
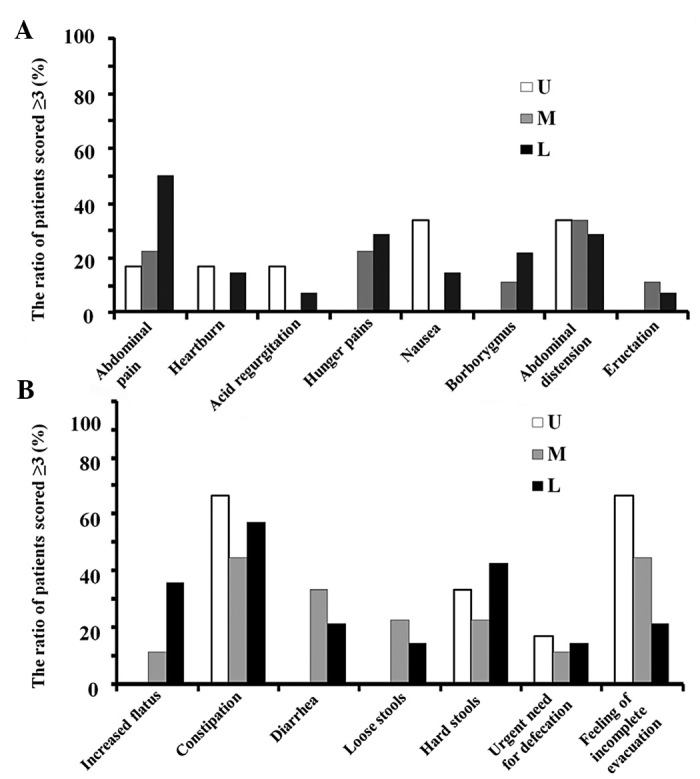
Ratio of patients with GI symptoms (≥mild discomfort) according to lesion location. Of the 29 patients that were evaluated for GI symptoms after ESD, 25 were scored ≥3 (≥mild discomfort) for at least one of the 15 GSRS items. GI, gastrointestinal; ESD, endoscopic submucosal dissection; GSRS, Gastrointestinal Symptom Rating Scale. U, upper third; M, middle third; L, lower third.

GI symptoms were evaluated in 29 of the 33 patients subjected to ESD. Of these 29 patients, 25 were scored ≥3 (≥mild discomfort) for at least one of the 15 GSRS items. The ratio of patients with GI symptoms (≥mild discomfort) according to lesion location is shown in Fig. 3. The lesion location was defined by anatomically dividing the stomach into three portions according to the Japanese classification of gastric carcinoma: the upper (U), middle (M) and lower (L) parts (17,18). Constipation (mean of U, M and L, 56%), a sense of incomplete evacuation (44%), hard stools (33%), abdominal distension (32%) and abdominal pain (30%) were reported in ≥30% of the patients irrespective of the lesion location. The incidences of nausea and a sense of incomplete evacuation were higher in the patients whose lesion was located in the U portion (33 and 67%, respectively) compared with those in the patients whose lesion was located in the M (0 and 44%, respectively) or L portions (14 and 21%, respectively). The incidence of upper abdominal pain was higher in the patients whose lesion was located in the L portion (50%) than in those whose lesion was located in the U (17%) or M portions (22%).

### Effect of rikkunshito on GI symptoms after ESD

**Figure 4.**
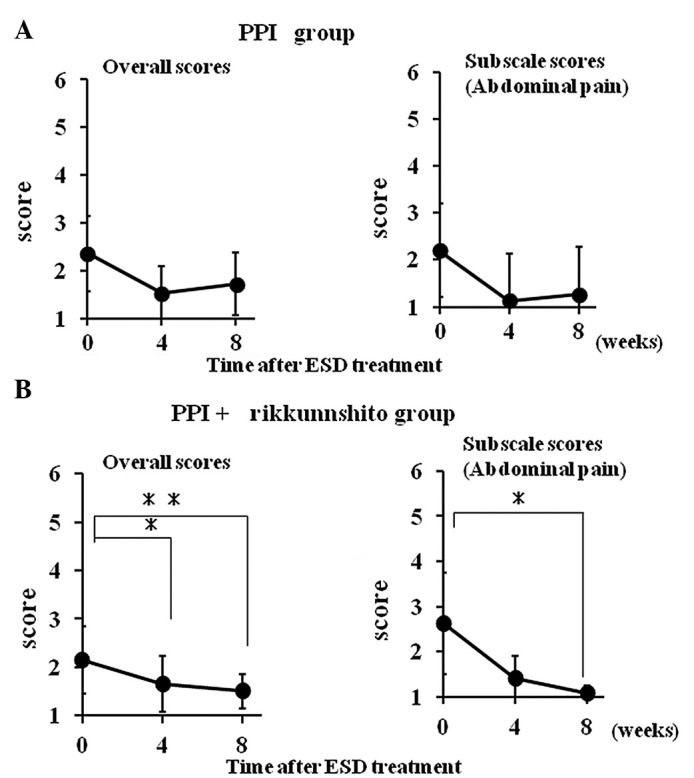
Changes in the overall scores and subscale GSRS scores (abdominal score) after treatment with (A) a PPI or (B) a PPI + rikkunshito. Closed circles indicate mean scores. ^*^P<0.05, ^**^P<0.01 vs. the score at baseline (Wilcoxon signed-rank test). GSRS, Gastrointestinal Symptom Rating Scale; PPI, proton pump inhibitor; ESD, endoscopic submucosal dissection.

**Figure 5.**
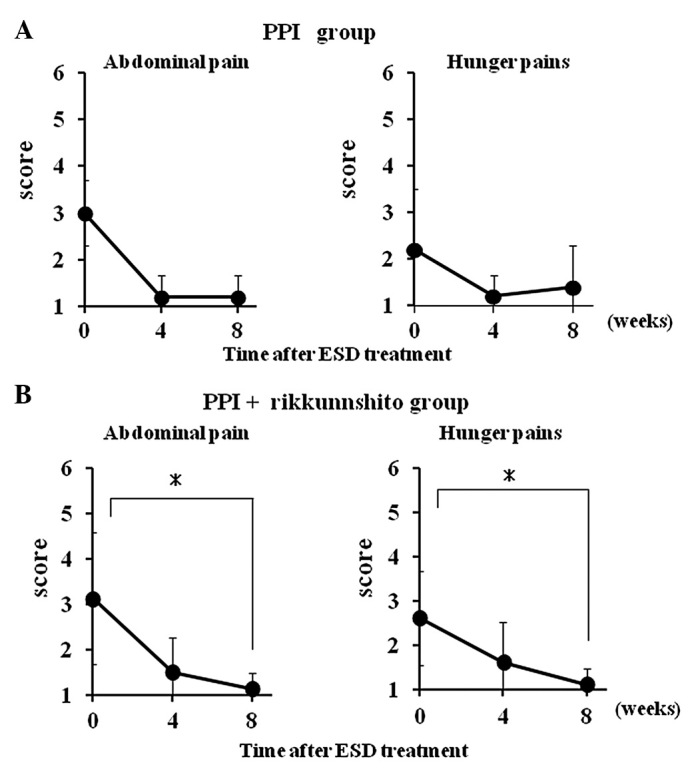
Changes of abdominal pain GSRS scores (abdominal and hunger pain) after treatment with (A) a PPI or (B) a PPI + rikkunshito. Closed circles indicate mean scores. ^*^P<0.05 vs. the score at baseline (Wilcoxon signed-rank test). GSRS, Gastrointestinal Symptom Rating Scale; PPI, proton pump inhibitor; ESD, endoscopic submucosal dissection.

Thirteen patients who scored ≥3 more than the average scores for abdominal pain or indigestion 6–8 days after ESD were included in the Step 2 study. The overall scores and the five subscale scores of GSRS after 4 and 8 weeks of treatment with a PPI alone (rabeprazole 20 mg/day, b.i.d, n=5) or a PPI (rabeprazole 20 mg/day, b.i.d, n=8) combined with rikkunshito (7.5 g/day, t.i.d) are shown in Table II. The overall scores and five subscale scores of GSRS did not change after 4 and 8 weeks of treatment with the PPI alone, while a significant improvement of overall GSRS scores was observed after 4 and 8 weeks of treatment with PPI plus rikkunshito (P=0.1313). Moreover, the mean abdominal pain score was significantly decreased after 8 weeks of treatment with PPI plus rikkunshito (Fig. 4), indicating the beneficial effects of rikkunshito, particularly against abdominal and hunger pains (Fig. 5).

## Discussion

The mean T-max 6–8 days after ESD was 75.4±13.6 min which was significantly longer compared with the mean T-max previously reported in healthy subjects (43.9±10.3 min) (16). This suggests that gastric emptying was delayed 6–8 days after ESD; however, gastric emptying beyond this operative period remains unknown. Furthermore, we examined the correlation between delayed gastric emptying and lesion location, and observed that the gastric emptying tended to be more delayed in patients whose lesion was located in the U portion [T-max (mean ± SD), 87.5±6.1] compared with those whose lesion was located in the M [T-max (mean ± SD), 75.0±12.2] or L portions [T-max (mean ± SD), 67.7±14.9] (data not shown). Normal gastric emptying is known to reflect a coordinated effort of the fundus, antrum, pyloric sphincter and duodenum (19). Moreover, the gastric emptying of solid and liquid foods is regulated by different mechanisms (19–21). Liquid emptying mainly depends on the gastric-duodenal pressure gradient derived from prolonged contraction of the fundus with less reliance on antral peristalsis and pyloric opening. By contrast, solids are initially retained selectively within the stomach until particles have been triturated to a size of <2 mm, and the solid emptying mainly depends on antral and pylorus actions. Since a liquid meal was used in the gastric emptying test, the action of the fundus is likely to have had a potent effect on gastric emptying. The U portion of the stomach includes the fundus. Therefore, gastric emptying is likely to be further delayed in patients whose lesion is located in the U portion compared with those whose lesion is located in the M or L portion.

GI symptoms associated with delayed gastric emptying were observed 6–8 days after ESD. In particular, constipation, a sense of incomplete evacuation and abdominal distension were observed in ≥30% of the patients. It is not clear whether delayed gastric emptying causes constipation, although voluntary suppression of defecation is known to delay gastric emptying in healthy subjects (22). Furthermore, constipation-predominant IBS patients have been reported to experience delayed gastric emptying more frequently compared with healthy controls or diarrhea-predominant IBS patients (23). Consequently, constipation may further delay gastric emptying.

The present study demonstrated that the incidence of upper abdominal pain was higher in the patients whose lesion was located in the L portion than in those whose lesion was located in the U or M portion. Since the L portion of the stomach includes the antrum and the pylorus, the L portion is subject to the effects of smooth muscle contraction/relaxation and bile acid regurgitation by antroduodenal coordination (19). Therefore, many patients with a lesion in the L portion appear to experience abdominal pain.

In the present study, we evaluated the effectiveness of rikkunshito against GI symptoms following ESD. The overall and mean abdominal pain GSRS scores were improved following treatment with a PPI plus rikkunshito but not after treatment with a PPI alone. In patients with PPI-refractory gastroesophageal reflux disease, treatment with PPI plus rikkunshito has been shown to improve upper GI symptoms, supporting the results of the present study (10). Since rikkunshito does not have the gastric anti-secretory effect of an acid reducer such as PPI 24, rikkunshito may improve abdominal pain by a mechanism different from that of PPI. Rikkunshito has various pharmacological actions such as a stimulatory effect on gastric emptying (25–27), regulation of ghrelin secretion (28–30) and protection of the gastric mucosa (31). Rikkunshito has been shown to improve upper GI symptoms via the stimulation of gastric emptying in functional dyspeptic (FD) patients (25) and in patients who had undergone pylorus-preserving gastrectomy (26). Moreover, hesperidine and atractylodin, which are ingredients of rikkunshito, have been found to improve delayed gastric emptying in rats following L-NNA-administration (27,32). Ghrelin is a digestive system hormone, originally identified in the stomach as the endogenous ligand for the growth hormone secretagogue receptor GHS-R1a (33). Ghrelin has a wide spectrum of biological functions including appetite stimulation, GI motility and gastric mucosal protection (34,35). Recently, rikkunshito was shown to enhance ghrelin secretion and the reactivity of its receptor (28,36). In particular, the plasma acyl-ghrelin concentration is increased in healthy volunteers and FD patients following treatment with rikkunshito (29,30). Thus, the stimulatory effect of gastric emptying and ghrelin secretion caused by rikkunshito plays a major role in the alleviation of GI symptoms. The improvement in abdominal pain following ESD may be due to these pharmacological effects. The effects of rikkunshito on gastric emptying and plasma ghrelin concentration in patients who have undergone ESD warrant further investigation.

In conclusion, ESD affects gastric emptying and is associated with upper GI symptoms such as abdominal pain and indigestion. Treatment with a PPI plus rikkunshito may improve the GI symptoms in patients who have undergone ESD when the GI symptoms are not improved by treatment with a PPI alone.

## Figures and Tables

**Table I.** Clinicopathological characteristics of early gastric cancer patients.

Characteristic	Value, n (range)
No. of patients	33
Age (years)	70 (56–79)
Gender (male/female)	25/8
Body mass index	23 (17–30)
ESD of gastric lesions	
Guideline criteria	11
Expanded criteria	7
Other	15
Lesion size (mm)	27 (7–70)
Length of the resected specimen (mm)	50 (18–90)
En bloc resection/piecemeal removal	33/0
Complete removal/incomplete removal	32/1
Lesion localization in the stomach	
Upper third	6
Middle third	11
Lower third	16
Lesion site in the stomach	
Anterior wall	5
Posterior wall	7
Lesser curvature	13
Greater curvature	7
Other	1

ESD, endoscopic submucosal dissection.

**Table II.** Overall scores and the five subscale scores of the GSRS after 4 and 8 weeks of treatment with a PPI alone or a PPI combined with rikkunshito.

Subscale score	PPI group (A)			PPI + rikkunshito group (B)		
	Baseline	4 weeks	8 weeks	Baseline	4 weeks	8 weeks
Reflux syndrome	1.50±0.61	1.40±0.65	1.30±0.45	1.63±0.64	1.06±0.18	1.13±0.35
Abdominal pain	2.20±0.51	1.13±0.18	1.27±0.37	2.63±1.13	1.42±0.50	1.08±0.15^a^
Indigestion syndrome	2.40±0.86	1.45±0.45	1.75±0.74	2.06±0.98	1.75±1.03	1.47±0.49
Diarrhoea syndrome	2.40±1.21	1.27±0.37	1.25±0.50	1.67±0.71	1.38±0.58	1.38±0.45
Constipation syndrome	3.27±1.40	2.40±1.67	3.08±1.60	2.75±1.38	2.67±1.55	2.50±1.47
Overall scores	2.35±0.79	1.53±0.56	1.73±0.66	2.15±0.69	1.65±0.58^a^	1.51±0.35^b^

a P<0.05,

b P<0.01 vs. baseline.

GSRS, Gastrointestinal Symptom Rating Scale; PPI, proton pump inhibitor.
